# The indirect role of orthorexia nervosa and eating attitudes in the association between perfectionism and muscle dysmorphic disorder in Lebanese male University students – results of a pilot study

**DOI:** 10.1186/s12888-023-04549-7

**Published:** 2023-01-20

**Authors:** Georges Merhy, Verginia Moubarak, Rabih Hallit, Sahar Obeid, Souheil Hallit

**Affiliations:** 1 School of Medicine and Medical Sciences, Holy Spirit University of Kaslik, P.O. Box 446, Jounieh, Lebanon; 2 Infectious Disease Department, Notre Dame des Secours University Hospital Center, Street 93, Byblos, Postal Code 3, Lebanon; 3 Infectious Disease Department, Bellevue Medical Center, Mansourieh, Lebanon; 4 Social and Education Sciences Department, School of Arts and Sciences, Lebanese American University, Jbeil, Lebanon; 5 Research Department, Psychiatric Hospital of the Cross, Jal Eddib, Lebanon; 6 Applied Science Research Center, Applied Science Private University, Amman, Jordan

**Keywords:** Muscle dysmorphia, Orthorexia nervosa, Perfectionism, Eating attitudes, Males, Lebanon

## Abstract

**Background:**

The literature highly concentrates on disorders related to body image among women but only minimally when it comes to the male population; hence, in order to provide general practitioners, and primary care physicians in general, and psychiatrists in particular, with additional information concerning muscle dysmorphia among male university students in Lebanon, this study seemed essential, and was therefore conducted to (1) identify the prevalence of MDD, and (2) evaluate the indirect effect of eating attitudes in general and orthorexia nervosa in particular, in the association between perfectionism and muscle dysmorphic disorder (MDD) among a sample of male university students.

**Methods:**

In this cross-sectional study conducted between September 2021 and May 2022, 396 male university students from multiple universities in Lebanon filled the online Arabic questionnaire.

**Results:**

The results showed that 26 (6.6%) of the participants had MDD. Orthorexia nervosa and eating attitudes mediated the association between perfectionism and MDD; higher perfectionism was significantly associated with higher ON and more inappropriate eating; higher ON and more inappropriate eating were significantly associated with higher MDD, whereas perfectionism had a significant total direct effect on MDD. The high prevalence of MDD among male university students in Lebanon implies further investigation on the national level in the country.

**Conclusion:**

Awareness campaigns among the university students could be adopted at the national level to increase the level of knowledge on the concepts of obsessive self-destructive perfectionism, orthorexia nervosa and muscle dysmorphia.

## Background

Muscle Dysmorphic Disorder (MDD), or bigorexia, is defined by the Diagnostic and Statistical Manual of Mental Disorders, 5th edition (DSM-5) as a body dysmorphic disorder that triggers a preoccupation with the idea that one’s body is too small or not muscular enough. It is considered to be one of the specifiers of body dysmorphic disorders [1]. People with body dysmorphic disorders typically present compulsory behaviors, which they sustain in the conviction that going through with them might help them achieve the body goal they desire [1]. These compulsions can be reflected by different actions, as it can range from spending hours in the gym, spending excessive amounts of money on ineffectual sports supplements, abnormal eating patterns or even sometimes by substance abuse [2].

The diagnosis of Body Dysmorphic Disorders (BDD) is based on many criteria which were addressed in the DSM-5. One of the main criteria that can help diagnose a patient with a body dysmorphic disorder is the preoccupation, with one or many perceived but minimal flaws in the person’s physique. Furthermore, performing repetitive behaviors like mirror checking, excessive grooming, reassurance- seeking or mental acts, like comparing his or her appearance with others, are also considered to be important aspects in the diagnosis of BDD. In addition to a continuous distress and impairment in social, occupational and other important areas of functioning, people with BDD should be screened for a concomitant eating disorder which should always be ruled out [1]. Muscle dysmorphia being a specifier of body dysmorphic disorders, another commonly found feature is the preoccupation of the individual with the idea that his body build is too small or insufficiently muscular [1]. Moreover, MDD can affect the lifestyle of individuals by triggering social isolation or by causing some impairment in social and occupational functioning owing to the extended hours spent exercising, the constant preoccupancy and focus on their diet, and the withdrawal from any other social, occupational, or recreational activities [3]. Some MD sufferers were reported to refuse social invitations or beach outings because of their fear of looking too small [3].

### Sociodemographic characteristics of MDD

BDD seems to affect a significant number of university students of various specialties. A study done on young adults majoring in biology, sport sciences and dietetics showed a prevalence of muscle dysmorphia of 5.9% [4]. When it comes to gender, while the medical literature discusses vastly the disorders of body image in women, especially in eating disorders [5], a recent growing interest in male body image research has only lately emerged, going from the fact that males desire a more muscular body and a stronger physique [6, 7]. Many variables including body mass, media influences, ideal body internalization, low self-esteem, body dissatisfaction, negative affect, perfectionism, and body distortion were identified through the medical literature as variables implicated in muscle dysmorphic disorders in women [8]. Other studies targeting different age groups in males, suggest that a higher risk of MD was associated with younger ages [9], but found no significant difference between low-risk and high-risk groups of gym users regarding their marital status, level of education, or Body Mass Index (BMI) [10]. Moreover, a higher risk of developing muscle dysmorphia (MD) was correlated with a higher frequency of training and higher numbers of competitions but not with longer durations of training during the week [10]. A study conducted on 3618 Australian adolescents also showed the independence of the prevalence MD from socioeconomic status (SES) [11].

### MDD and perfectionism

Perfectionism can be defined as a multidimensional measure, it is the tendency to claim of others or of oneself an extremely high or even flawless level of performance, more than what is needed by the situation [1]. Perfectionism is not considered a psychological disorder in itself; however, it is linked to anxiety and other mental health issues, such as obsessive–compulsive disorder (OCD) [12], depression, anxiety, and eating disorders [1]. Noting that there are 5 main styles of perfectionism: self-oriented perfectionism, socially prescribed perfectionism, other-oriented perfectionism, overt perfectionism, and covert perfectionism [13]. As previously mentioned, perfectionism constitute one of the variables affecting muscle dysmorphia in women [8]; hence, a similar correlation could also be questioned in men. Indeed, in a meta-analysis which was aimed at evaluating the association between perfectionism and MDD, and which included 5880 participants, both men and women, from 31 studies (21 of them targeting men, 1 targeting women, and the remaining targeting both), perfectionism was found to be positively correlated with muscle dysmorphia [14]. Moreover, perfectionism was associated with concurrent body dysmorphic disorder symptoms among adolescents [14]. This association remained significant even when controlling over other confounding factors such as anxiety and depression [14]. Other results suggest that susceptibility to Muscle Dysmorphia and Eating disorders depend on pre-existing perfectionistic attitudes, especially that of socially prescribed perfectionism [15].

### MDD and disordered eating

Eating disorders are defined by the DSM-5 as a persistent disturbance of eating [1]. These behavioral conditions can be associated with distressing thoughts or emotions which can affect the individual’s physical, psychological, and social function [16]. These eating attitudes can be explained by inappropriate thoughts, feelings, beliefs, and relationship an individual might experience with food, which can then influence his nutritional choices, his behavioral and consequently his health status [17]. A significant positive relationship between Muscle Dysmorphic Disorder Inventory (MDDI) and Eating Attitudes Test (EAT-40) in both professional and recreational bodybuilders was recently found [18]. Muscle Dysmorphia in men seems to have parallel features to eating disorders in women, the mutual component between eating disorders (EDs) and muscle dysmorphia being body dissatisfaction [8]. Indeed, males with eating disorders appear to possess higher desires for a more muscular shape, and therefore tend to perform body weight-related sports [18].

Orthorexia Nervosa (ON) is described as a pathological fixation on healthy food intake in addition to an excessive worrying with disturbing thoughts concerning healthy dietary consumption [19, 20]. The healthy eating obsession is pursued by ON sufferers through a strict diet centered on food quality and not quantity, demonstrating unrealistic concern over food selection, preparation and eating [21]. In Lebanon, one of the eating disorders that seem to have an unexpected high prevalence among the population, is Orthorexia nervosa (ON). A large number of the Lebanese population therefore appears to be preoccupied with healthy behavior and nutrition [21]. A previous study, conducted among Italian university students, shed the light on the possible relationship between ON, MD and eating disorders. In fact, the conditions were overlapping in the co-presence of ON, MD and EDs traits [22]. But further research and understanding of the effect of orthorexia nervosa and eating attitudes on the symptomatology of muscle dysmorphia is still needed.

#### Perfectionism, eating attitudes, orthorexia nervosa and MDD

In terms of relationship with eating behaviors, a systematic study, reviewing 55 papers published between 1990 and 2005, highlighted the relation between perfectionism and the diagnosis of eating disorders, anxiety disorders, and mood disorders [23]. Self-oriented perfectionism, which is one of the types of perfectionism that were previously stated, was found to be more specific to eating disorders in particular, than it is to depressive or anxiety disorders [24]. On the other hand, inappropriate eating behaviors including orthorexia nervosa, anorexia nervosa, and bulimia nervosa, shares similarities with regards to perfectionism, body image attitudes, and attachment style [26]. Moreover, having a history of an eating disorder seems to highly predict orthorexia nervosa. Within the literature, there is a continuing debate about the classification of orthorexia nervosa as a separate disorder [25], an alternative of an already established eating disorder or obsessive compulsive disorder (OCD) [26]. Some researchers propose that it could be a precursor for, or a residual of an eating disorder [27]. The results of a cohort study suggested that ON may be a continuum of anorexia nervosa and bulimia nervosa, where a person switches from an obsession with the quantity to the quality of food [27]. Therefore, these disorders might be on the same spectrum of disordered eating [26]. However, a recent study showed that ON does not sufficiently predict OCD symptoms [28].

The association between orthorexia nervosa and perfectionism has been rarely studied. Adherence to perfect rules in life is a predictor of mental illness and eating disorders [29]. Higher perfectionism was associated with more orthorexia nervosa in two studies [30–32]. Recent findings showed that all dimensions of perfectionism were positively associated with following strict eating rules, and namely, ON [33]. Valente et al. demonstrated the same findings and concluded that since perfectionism is focused around the desire to control one’s life events, ON might be a trial to control one’s own nutrition in life [34].

Eating disorder and muscle dysmorphic share lots of similarities; obsession in increased muscle mass may lead to eating disorders [35]. One study found a negative association between MD and ON [36], while another one found a positive association between ON tendencies and perceived muscularity [37]. Other researchers determined that persons at risk of MD might also be at risk or ON, general disordered eating or both [38]. Of note, the literature previously highlighted the role of perfectionism as a mediator of the association between orthorexia nervosa and excessive exercising [27], yet no light was given for the mediating role of ON. Further investigation is still needed as for the mediating effect of orthorexia nervosa and other eating attitudes on the correlation between perfectionism and muscle dysmorphia.

### The present study

The Lebanese fitness industry has been growing significantly. The industry witnessed a growth of 20%, between 2010 and 2015, which proves the interest of the Lebanese population in general and especially the young adults in fitness [39]. However, since 2019, the country witnessed significant economic crises with income losses, inflation, and weakening of the Lebanese currency that drove more than half of the citizens below the poverty line [40]. Thus, there have been limited studies assessing the impact of such events on the modification of sociodemographic characteristics including socioeconomic status, physical activity and financial burden that might be affecting MDD among the young Lebanese males. The literature highly concentrates on disorders related to body image among women but only minimally when it comes to the male population [5]; hence, in order to provide general practitioners, and primary care physicians in general, and psychiatrists in particular, with additional information concerning muscle dysmorphia among male university students in Lebanon, this study seemed essential, and was therefore conducted to (1) identify the prevalence of MDD, and (2) evaluate the indirect effect of eating attitudes in general and orthorexia nervosa in particular, in the association between perfectionism and MDD among a sample of male university students.

## Methods

### Study design and sampling

This cross-sectional study was carried out between November 2021 and May 2022; 396 university students were recruited through convenience sampling through several universities in Lebanon’s governorates. At first, the research team contacted university students they know, who received the online link to the survey (created on Google forms). Those who agreed to participate were then asked to forward the link to other students they know in their university or in another one, which explains the snowball sampling technique followed in the data collection. Students who agreed to participate in the study were directed to the consent form, which contained a paragraph that explained the purpose of the current study, ensured anonymity of the participant, and the voluntariness of consent to research. Once the student gave his/her consent, he/she was directed to the questionnaire. All participants responded willingly to the survey. There were no fees for participating in the study. All university students over the age of 18 were eligible to participate. Excluded were those who refused to complete the survey.

### Minimal sample size

A minimal sample of 124 was deemed necessary using the formula suggested by Fritz and MacKinnon [41] to estimate the sample size: $n=\frac{L}{f2}+k+1$, where f = 0.26 for moderate effect size, L = 7.85 for an α error of 5% and power β = 80%, and k = 7 variables to be entered in the model.

### Measures

The first part of the questionnaire involved socio-economic features including age, marital status, and household crowding index reflecting the socioeconomic status of the family, which was calculated by dividing the number of persons in the house by the number of rooms in the house excluding the bathrooms and kitchen [42]. Body Mass Index (BMI) was calculated from self-reported height and weight. The physical activity index was calculated by multiplying the intensity by the frequency by the time of physical activity [43]. Participants were asked to rate the financial burden using one question on a scale from 1 (low) to 10 (high), with 10 referring to the most overwhelming pressure. The second part of the questionnaire included the following scales:

- *Muscle Dysmorphic Disorder Inventory (MDDI)* is a 13-item questionnaire that contains three subscales directly related to MD: “drive for size” used to measure the desire to increase muscle mass, “appearance intolerance” used to measure the desire to lose fat mass, and “functional impairment” used to measure avoidance of social situations [44]. Respondents rate statements on a 1 (never) to 5 (always) scale. The three subscales’ scores are: Drive for Size (DFS, 5 items, range 5–25), Appearance Intolerance (AI, 4 items, range 4–20), and Functional Impairment (FI, 4 items, range 4–20). These three scores yield a total MDDI score ranging from 13 to 65; participants scoring 39 and more were classified as having MDD symptoms [44]. (Cronbach’s alpha for the total score in this study = 0.81).
- *Düsseldorf Orthorexia Scale (DOS)* includes ten items, to which respondents answer on a four-point Likert scale where 1 = never, 2 = rarely, 3 = often, and 4 = always [45]. The Arabic version of the DOS seems to be a structurally valid and internally consistent questionnaire measuring orthorexic eating behavior in Lebanese adolescents [46] and adults [47]. Higher scores indicate higher orthorexia nervosa. (Cronbach’s alpha in this study = 0.87).
- *Eating Attitudes Test (EAT-26)* is used to identify the presence of “eating disorder risk” based on attitudes, feelings and behaviors related to eating. The scale has three subscales: Dieting, Bulimia and Food Preoccupation, and Oral Control [48]. A score of 20 or above is used as a clinical cut-off and indicates inappropriate eating behaviors. All subscales can be added to give a total score, or each subscale can be used independently. The EAT-26 has been validated and translated into Arabic [49]. (Cronbach’s alpha in this study = 0.97).
- *Big Three Perfectionism Scale* is composed of 16 items, scored on a five-point Likert scale (1 = strongly disagree to 5 = strongly agree) [50]. It yields three subscales’ scores: rigid perfectionism, self-critical perfectionism, and narcissistic perfectionism. Higher scores reflect higher perfectionism in the three aspects. In this study, the Cronbach’s alpha values for the three scores were as follows: rigid perfectionism (α = 0.90), self-critical perfectionism (α = 0.88) and narcissistic perfectionism (α = 0.84).

### Translation procedure

The forward and backward translation method was applied to non-validated scales [51]. The English version was translated to Arabic by a Lebanese translator who was completely unrelated to the study. Afterwards, a Lebanese psychologist with a full working proficiency in English, translated the Arabic version back to English. The initial English version and the second English version were compared to detect and later eliminate any inconsistencies.

### Statistical analysis

Data analysis was performed with SPSS software version 25 (IBM, New York, NY, USA). The MDDI score followed a normal distribution (skewness (=0.733) and kurtosis (=0.410) values between -1 and +1 [52].The Pearson test was used to correlate two continuous variables. The Cronbach’s alpha value was computed to assess scales’ reliability. The PROCESS SPSS Macro version 3.4, model four was used to check for a possible mediating effect of disordered eating attitudes and orthorexia nervosa in the association between perfectionism and MDDI. A significant mediation was determined if the confidence interval (CI) around the indirect effect did not include zero [53]. Covariates that were included in the mediation model were those that had a p<0.25 in the bivariate analysis. Significance was set at *p* < 0.05.

## Results

A total of 396 young male adults completed the questionnaire; their mean age was 25.39 years, with 79% single. The results showed that the mean MDD score was 26.88 ± 7.64, with 26 (6.6%) of the participants showing MDD (scores ≥ 39). Furthermore, the mean EAT score was 33.14 ± 23.17, with 254 (64.1%) of the sample showing inappropriate eating attitudes (scores ≥ 20). The mean DOS score was 20.31 ± 6.30, with 58 (14.6%) students having possible orthorexia nervosa tendencies, whereas 40 (10.1%) had ON tendencies. Other characteristics of the participants are summarized in Table 1.

**Table 1:** Sociodemographic and other characteristics of the participants (*N* = 396)

Variable	N (%)
Marital status	
Single	313 (79.0%)
Married	83 (21.0%)
**Mean ± SD**	
Age (in years)	25.39 ± 4.96
Body Mass Index (kg/m^2^)	24.46 ± 3.51
Household crowding index (person/room)	0.81 ± 0.40
Physical activity index	36.65 ± 23.77
Financial burden	5.44 ± 2.97
Eating attitudes test score	33.14 ± 23.17
Orthorexia nervosa score (DOS score)	20.31 ± 6.30
Muscle Dysmorphic Disorder score	26.88 ± 7.64

### Bivariate analysis of factors associated with MDD

Higher financial burden (*r*=0.37), orthorexia nervosa (*r*=0.41), rigid perfectionism (*r*=0.31), self-critical perfectionism (*r*=0.37), and narcissistic perfectionism (*r*=0.25) were significantly associated with higher MDDI scores (Table 2).

**Table 2:** Correlation of continuous variables with the MDD score

Variable	1	2	3	4	5	6	7	8	9	10
1. MDD	1									
2. Age	-0.002	1								
3. Household crowding index	0.05	**-0.1**	1							
4. Physical activity index	-0.03	-0.08	**-0.21**	1						
5. Financial burden	**0.37**	**0.11**	0.09	**-0.20**	1					
6. Eating attitudes	-0.07	-0.01	**-0.20**	**0.24**	**-0.30**	1				
7. Orthorexia nervosa	**0.41**	-0.03	0.07	0.02	**0.23**	**-0.24**	1			
8. Rigid perfectionism	**0.31**	**-0.11**	**0.22**	**-0.21**	**0.37**	**-0.56**	**0.40**	1		
9. Self-critical perfectionism	**0.37**	0.02	**0.13**	**-0.23**	**0.37**	**-0.47**	**0.36**	**0.72**	1	
10. Narcissistic perfectionism	**0.25**	0.04	0.03	-0.09	**0.24**	**-0.35**	**0.47**	**0.61**	**0.58**	1

Numbers in bold indicate significant *p*-values

*MDD* Muscle Dysmorphic Disorder

### Mediation analysis

**Fig. 1:**
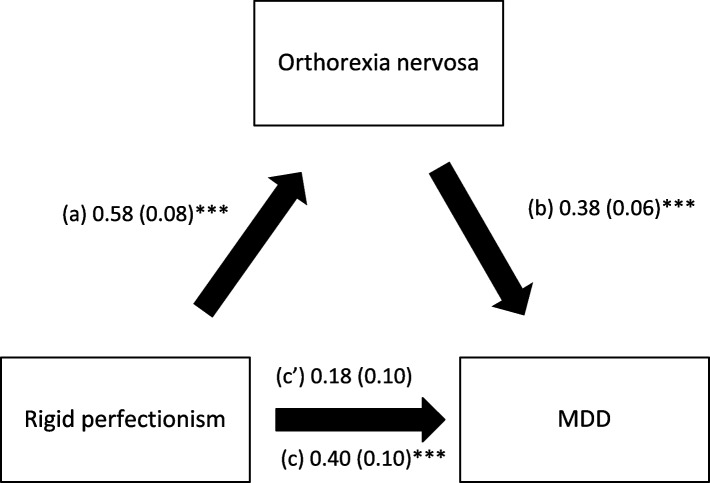
**a** Relation between rigid perfectionism and orthorexia nervosa; (**b**) Relation between orthorexia nervosa and MDD; (**c**) Total effect of rigid perfectionism on MDD; (**c**’) Direct effect of rigid perfectionism on MDD. Numbers are displayed as regression coefficients (standard error). ***p<0.001. MDD = Muscle Dysmorphic Disorder

**Fig. 2:**
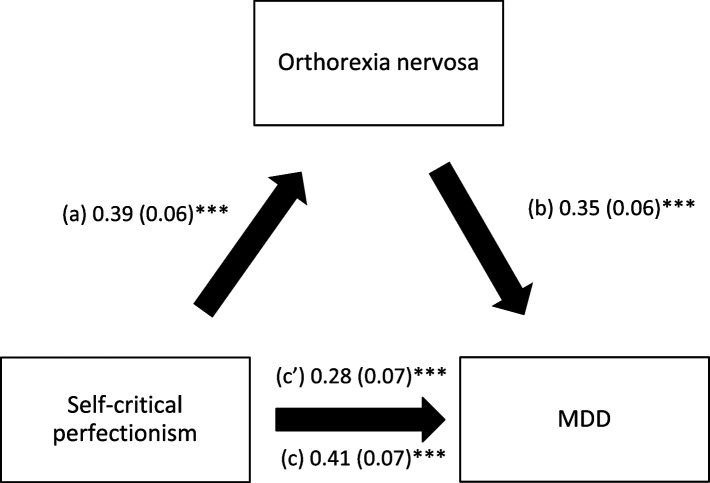
**a** Relation between self-critical perfectionism and orthorexia nervosa; (**b**) Relation between orthorexia nervosa and MDD; (**c**) Total effect of self-critical perfectionism on MDD; (**c**’) Direct effect of self-critical perfectionism on MDD. Numbers are displayed as regression coefficients (standard error). ***p<0.001

**Fig. 3:**
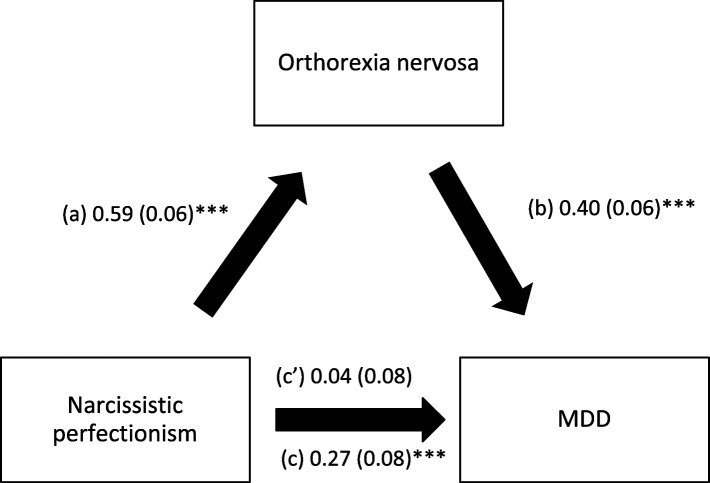
**a** Relation between narcissistic perfectionism and orthorexia nervosa; (**b**) Relation between orthorexia nervosa and MDD; (**c**) Total effect of narcissistic perfectionism on MDD; (**c**’) Direct effect of narcissistic perfectionism on MDD. Numbers are displayed as regression coefficients (standard error). ***p<0.001

Orthorexia nervosa mediated the association between the three types of perfectionism and MDD; higher perfectionism was significantly associated with higher ON; higher ON was significantly associated with higher MDD, whereas perfectionism had a significant total direct effect on MDD (Table 3, Figs. 1, 2 and 3).

**Table 3:** Mediation analysis: Direct and indirect effects of perfectionism and MDD, taking orthorexia nervosa and eating attitudes as mediators

	**Direct effect**			**Indirect effect**		
	**Effect**	**SE**	***p***	**Effect**	**SE**	**95% BCa**
Model 1: orthorexia nervosa as the mediator						
Rigid perfectionism	0.18	0.10	0.064	0.22	0.05	0.13; 0.33^a^
Self-critical perfectionism	0.28	0.07	< 0.001	0.14	0.04	0.07; 0.21^a^
Narcissistic perfectionism	0.04	0.08	0.640	0.24	0.05	0.15; 0.33^a^
Model 2: eating attitudes as the mediator						
Rigid perfectionism	0.58	0.11	< 0.001	-0.18	0.06	-0.30; -0.07^a^
Self-critical perfectionism	0.51	0.08	< 0.001	-0.10	0.03	-0.17; -0.03^a^
Narcissistic perfectionism	0.33	0.08	< 0.001	-0.05	0.03	-0.11; 0.001

^a^Indicates significant mediation; *Direct effect* Effect of perfectionism on MDD in the absence of the mediator, *Indirect effect* Effect of perfectionism on MDD in the presence of the mediator, *SE* Standard Error, *BCa* Bootstrap Confidence Interval

**Fig. 4:**
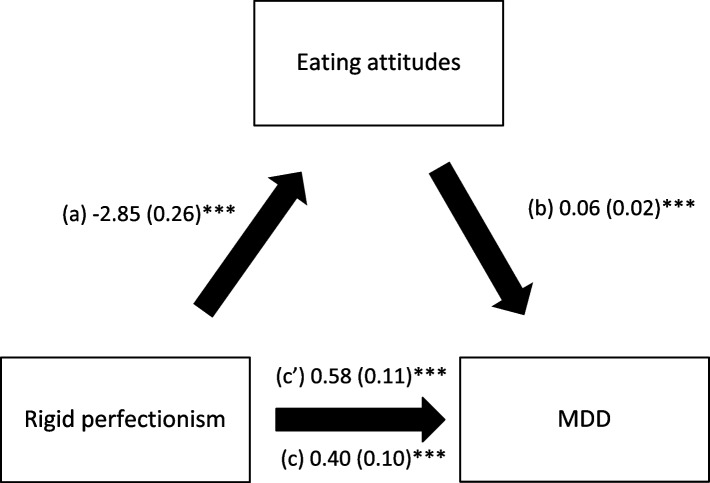
**a** Relation between rigid perfectionism and eating attitudes; (**b**) Relation between eating attitudes and MDD; (**c**) Total effect of rigid perfectionism on MDD; (**c**’) Direct effect of rigid perfectionism on MDD. Numbers are displayed as regression coefficients (standard error). ***p<0.001

**Fig. 5:**
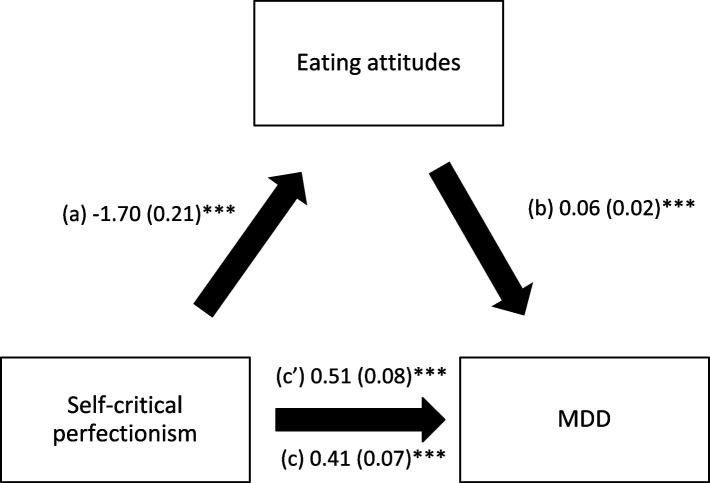
**a** Relation between self-critical perfectionism and eating attitudes; (**b**) Relation between eating attitudes and MDD; (**c**) Total effect of self-critical perfectionism on MDD; (**c**’) Direct effect of self-critical perfectionism on MDD. Numbers are displayed as regression coefficients (standard error). ***p<0.001

Eating attitudes mediated the association between rigid and self-critical perfectionism and MDD only; higher perfectionism was significantly associated with more appropriate eating; higher eating attitudes (more inappropriate eating) were significantly associated with higher MDD, whereas rigid and self-critical perfectionism had a significant total direct effect on MDD (Table 3, Figs. 4 and 5).

## Discussion

### Prevalence

Based on the MDDI scale, the results of our study showed that 6.6% of the participants had MDDI with a mean score of 26.88 ± 7.64. According to the International Obsessive-Compulsive Disorder (OCD) Foundation, the numbers suggest that about 0.5% of men in general may meet criteria for MD [7]; thus, the point prevalence of the male university students in Lebanon is considerably higher. Our research was conducted among university students, and the expected prevalence is lower in samples from general community and university students compared to bodybuilding or weightlifting groups [54]; hence, these findings might be alarming and suggest further studies on the national level. Furthermore, this increase in the prevalence might be due to the lack of awareness among the young generation during the phase of the economic crises. The financial burden caused by this crisis can also be preventing individuals with MDD from being detected and later supported with psychological therapies which also favors this increase.

### Mediation analysis

Our results shows that eating attitudes mediated the association between rigid and self-critical perfectionism and MDD only. In a previous study, higher self-critical and rigid perfectionism were correlated with higher eating disorders and with higher life dissatisfaction, thus worsening the psychological illness [55, 56]. Narcissistic perfectionism, linked to grandiosity, is often associated with higher life satisfaction [55, 56]. From here these findings and ours share similarities in terms of the independency of the narcissistic perfectionism from eating attitudes. Therefore, eating attitudes and inappropriate eating can mediate the effect of self and rigid perfectionism in individuals prone to the sensation of dissatisfaction and hence trigger more symptoms of muscle dysmorphia, which is also by definition a state of dissatisfaction of the muscle size.

Our findings also suggest that orthorexia nervosa might mediate the association between the three types of perfectionism and MDD. No previous study in the literature highlighted such mediation. Of note, previous study had tackled the mediating effect of perfectionism on excessive exercising among orthorexic individuals [57]. Additionally, another research suggested a different kind of mediation, where compulsive exercise mediated the effect of perfectionism on eating pathologies [58]. Hence, the triad ON, perfectionism, and MDD is also repeating itself in our study with a different mediation pathway; however, in all of the three studies the concepts are superimposable to the triad of: perfect nutrition, perfect behaviors, perfect muscled body. The overlapping of these concepts and the mediating effect of ON could be self-explanatory by the simple fact that ON is defined as healthy food obsession, and hence perfect eating comes to stress any perfectionist behavior, independently of its subtype, consequently worsening MDD symptomatology.

### Direct effect of perfectionism on MDD

Our findings showed that perfectionism (both rigid and self-critical perfectionism) had a significant total direct effect on MDD, consistent with the positive correlation between perfectionism and the symptomatology of muscle dysmorphia discussed in a previous systematic review and meta-analysis [54]. Moreover, according to Rica et al., body dissatisfaction in males is associated with higher prevalence of muscle dysmorphia symptomatology, appearance-oriented perfectionism, and to compulsive exercise [59]. Therefore, the increase in the prevalence of MDD with perfectionism, which was found in our study, could be explained by the urge for possessing a perfect image, which triggers a continuous reinforcement for exercising while keeping a constant ideation of “not enough” muscle yet, “not perfect enough image yet”. Additionally, most BDD sufferers seem to indeed oversee their flaws and seek to blend with what is known as ‘norms’; this might be due to the fact that they seek perfectionism in their own appearances [14].

### Perfectionism and eating attitude/orthorexia nervosa

This study also highlights that higher perfectionism is significantly associated with more appropriate eating. Our findings oppose the results of a Portuguese study where perfectionism was associated with disordered eating attitudes [59]. In 1995, Hewitt, Flett, & Ediger suggested in their study two models, for the effect of perfectionism on eating attitudes: perfectionist individuals in the first model fear the idea of their bodies not meeting the strict evaluative criteria they put on themselves and the environmental standards and hence will go towards disordered eating as a trial to satisfy their impulsive thoughts; while individuals of the second model are more affected by the fear of doing mistakes which will not allow them to have imperfect diets [60]. We suggest that our findings follow the second model where the fear of imperfection and the avoidance of mistakes contributed to better eating attitudes.

This study highlights the idea that higher perfectionism was significantly associated with higher ON; which was indeed recently stated in the literature. For instance, in their study Yung and Tabri also found an indirect positive association between perfectionism and ON symptoms. Their study suggests that an explanation of this association might be linked to the fact that when an individual is aiming to eat in a healthy manner, perfectionism, in such cases, might emphasize ON symptoms indirectly by favoring a health-focused self-concept [61]. And hence “perfect healthy food” and perfectionist behaviors are rhyming ideations and the presence of one could increase the risk of the coexistence of the other.

### Eating attitude/Orthorexia nervosa and MDDI

Our results suggest that more detrimental eating attitudes (more inappropriate eating) were significantly associated with higher MDD. These findings are similar to those of the meta-analytic study reviewing 39 articles, which also reflected the presence of a significant positive correlation between muscle dysmorphia and eating disorders symptomatology [62]. This meta-analysis contributed the reason behind this association to the fact that bigger muscle mass could not be achieved by exercising only, but additionally needs a protein rich dietary [62]. Therefore, our findings might be explained by the inclination of individuals with MDD towards restricted diets, that are richer in proteins (which are required for the muscle growth), and poorer in other nutrients (which are required for having a healthy body). This unbalanced diet, rich in only one of these elements, might therefore help the individuals to reach their “MDD goal” more rapidly and to reinforce it, while putting the health of the individual at risk. For instance, high-protein diets can have risk cholesterol precipitation in the urinary tract [63]. Moreover, a study conducted on 400 university students in Turkey also affirmed that eating attitudes are associated with bigorexia nervosa [54]. This study also suggests that an improvement of the eating attitudes occurred with bigger muscle masses [54]. So, this might give an insight on the potential explanation of this association; in fact, the body dissatisfaction found with higher MDD scores, and worse muscle dysmorphia symptomatology, might be the psychological trigger behind the alteration of eating attitudes, and their deviation towards unhealthy nutritional habits. Nonetheless, further investigation is still needed to assess the reversibility of this behavior after the improvement of muscle dysmorphia.

Our results showed that higher ON was significantly associated with higher MDD, which comes in line with the results of the study of Goodale et al. conducted on university students, which also affirms the significant positive correlation between muscle dysmorphia and orthorexia nervosa [36]. Other studies have also shed the light on the fact that higher ON tendencies could be associated with higher tendencies of MDD [36]. Based on this study, this type of association might be due to the fact that the symptomatology of ON is positively correlated with perceived muscularity, while being negatively correlated with body fat perception; and since both muscularity and low body fat are affected by exercises, the healthy eating obsession in ON might consequently develop into an additional exercise obsession and MDD [36].

### Limitations

Among the limitations of this study is the selection bias during data collection due to the undetermined refusal rate and the snowball technique used; therefore, the results are not generalizable. Muscle dysmorphia’s diagnosis in this study is based on self-reported symptoms without a psychiatrist’s examination for each student, which may result in under or even overestimation at a certain point in the number of students with this disorder. In addition, no exclusion criteria were implemented, in which those with preexisting conditions such as anorexia or other psychiatric disorder. A residual confounding bias might also be possible since many factors associated with the dependent variable were not included. The variables of interest (the dependent variable and the independent and mediator variables) should have a linear relationship. In this study, the correlation analysis only partially satisfies this assumption. On the other hand, the study only included male participants, so we cannot assume a general relationship between muscle dysmorphia and perfectionism. The questionnaires was not administered in a counterbalanced order to manage the order and sequence effect. An information bias is also possible since the answers of the students might not be totally accurate. Adding to that, some of the used scales have not been validated in Lebanon (such as the MDDI). Finally, due to its cross-sectional design, causation cannot be inferred. The financial burden of the participants was only assessed using a single self-reported question in this study and could be further evaluated by adding a more detailed and standardized questionnaire in further studies.

## Conclusion

This study provides insights on MDD and its correlated factors including financial burdens, perfectionism, eating attitudes, and orthorexia nervosa. In our study, we concluded that we have a prevalence of muscle dysmorphia of 6.6%, which is high in Lebanon compared to other regions. On the other hand, our study highlighted the indirect relation between perfectionism and muscle dysmorphia, through eating attitudes and orthorexia nervosa. Awareness campaigns among university students could be adopted at the national level to increase the level of knowledge on the concepts of obsessive self-destructive perfectionism, orthorexia nervosa and muscle dysmorphia. Further studies are recommended to be conducted in a country like Lebanon regarding muscle dysmorphia to investigate more the reasons behind the increased prevalence of muscle dysmorphia.

## Data Availability

The datasets generated and/or analysed during the current study are not publicly available due to restrictions from the ethics committee but are available from the corresponding author on reasonable request.
